# Supplementary material for: The discovery of novel noncoding RNAs in 50 bacterial genomes
Source: Nucleic Acids Res. 2024 Apr 22;52(9):5152–65. doi: 10.1093/nar/gkae248 (PMC11109978; doi:10.1093/nar/gkae248)
Supplement: gkae248_Supplemental_Files [file gkae248_supplemental_files.zip › Narunsky 50 Genomes RNA Motifs Files.zip!/Publication/63-L_buccalis/Figures/LRC-63-5-NC_013192_1_IGR_50_1979947_1980269.sto.pdf]

LRC-63-5-NC_013192_1_IGR_50_1979947_1980269.sto.r2r

5′-UAUCUUGGAUAUAAGAUUGAAGGUGUAGUUUUUUUAUUAAUAUAAAGAAAAUAAAACUAGAGAAUUUUUUUGUGAACAAGGAAAAUCAGCAGGUAAAGAUACUUOO
